# Microbes as carbendazim degraders: opportunity and challenge

**DOI:** 10.3389/fmicb.2024.1424825

**Published:** 2024-08-14

**Authors:** Yi Zhou, Tianyue Wang, Liping Wang, Pengfei Wang, Feiyu Chen, Pankaj Bhatt, Shaohua Chen, Xiuming Cui, Ye Yang, Wenping Zhang

**Affiliations:** ^1^Key Laboratory of Sustainable Utilization of Panax Notoginseng Resources of Yunnan Province, Faculty of Life Science and Technology, Kunming University of Science and Technology, Kunming, Yunnan, China; ^2^State Key Laboratory for Conservation and Utilization of Subtropical Agro-bioresources, Guangdong Province Key Laboratory of Microbial Signals and Disease Control, Integrative Microbiology Research Centre, South China Agricultural University, Guangzhou, China

**Keywords:** carbendazim, toxicity, microbial degradation, biodegradation pathway, bioremediation

## Abstract

Carbendazim (methyl benzimidazol-2-ylcarbamate, CBZ) is a systemic benzimidazole carbamate fungicide and can be used to control a wide range of fungal diseases caused by *Ascomycetes*, *Basidiomycetes* and *Deuteromycetes*. It is widely used in horticulture, forestry, agriculture, preservation and gardening due to its broad spectrum and leads to its accumulation in soil and water environmental systems, which may eventually pose a potential threat to non-target organisms through the ecological chain. Therefore, the removal of carbendazim residues from the environment is an urgent problem. Currently, a number of physical and chemical treatments are effective in degrading carbendazim. As a green and efficient strategy, microbial technology has the potential to degrade carbendazim into non-toxic and environmentally acceptable metabolites, which in turn can dissipate carbendazim from the contaminated environment. To date, a number of carbendazim-degrading microbes have been isolated and reported, including, but not limited to, *Bacillus*, *Pseudomonas*, *Rhodococcus*, *Sphingomonas*, and *Aeromonas*. Notably, the common degradation property shared by all strains was their ability to hydrolyze carbendazim to 2-aminobenzimidazole (2-AB). The complete mineralization of the degradation products is mainly dependent on the cleavage of the imidazole and benzene rings. Additionally, the currently reported genes for carbendazim degradation are MheI and CbmA, which are responsible for breaking the ester and amide bonds, respectively. This paper reviews the toxicity, microbial degradation of carbendazim, and bioremediation techniques for carbendazim-contaminated environments. This not only summarizes and enriches the theoretical basis of microbial degradation of carbendazim, but also provides practical guidance for bioremediation of carbendazim-contaminated residues in the environment.

## 1 Introduction

**Figure 1:**
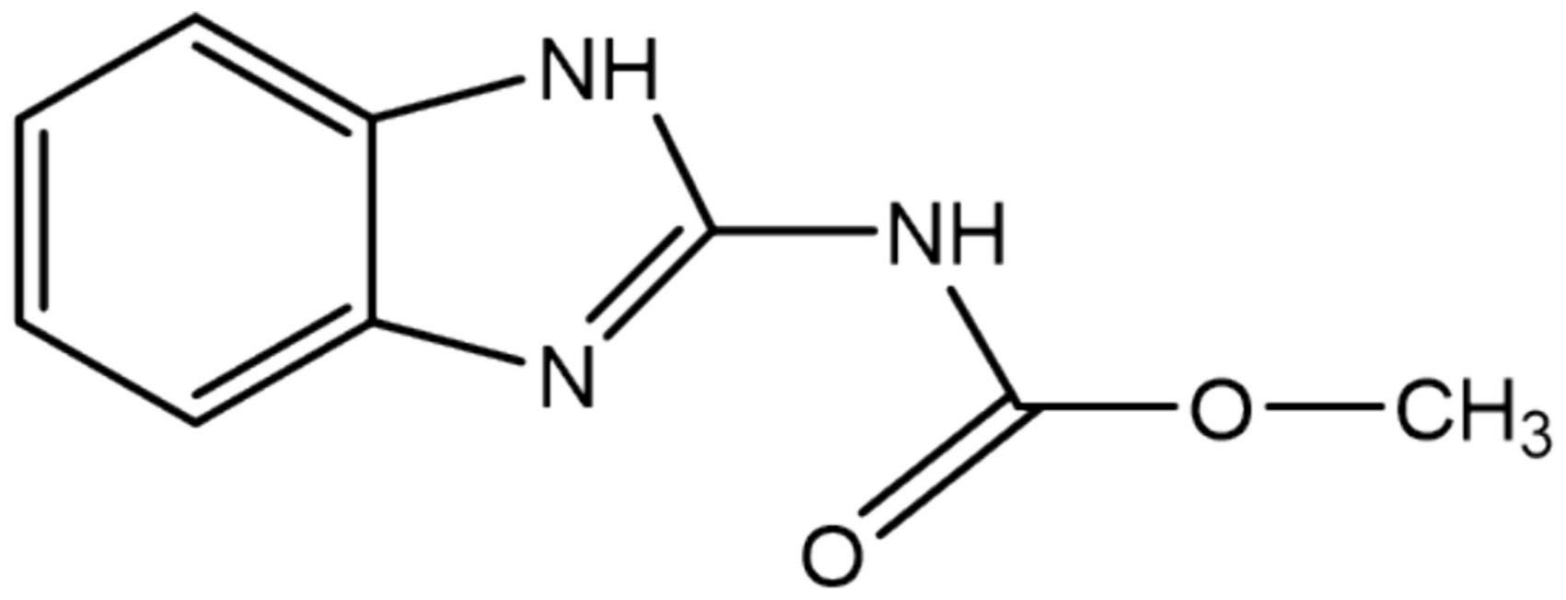
Structural formula of carbendazim.

Fungal pathogens cause significant damage to crops every year during crop production, which results in reduced crop yields, reduced nutritional value and significant economic losses. Thus, the usage of fungicides is growing in popularity as a way to prevent these losses (Singh et al., 2016). However, while fungicides are widely used, their residues also seriously jeopardize human health and ecological safety (Huang et al., 2018). Carbendazim (CBZ), also known as Methyl 1H-benzimidazol-2-ylcarbamate, is a benzimidazole, specifically 2-aminobenzimidazole with a methoxycarbonyl group substituting the primary amino group. It has the chemical formula C_9_H_9_N_3_O_2_, a structural formula shown in Figure 1, and a molecular weight of 191.19 (Bai et al., 2017). CBZ is a broad-spectrum systemic fungicide that is used to control various fungal diseases in agriculture, horticulture, and forestry. The antifungal mechanism of CBZ is by interfering with the synthesis and function of the fungal cell wall, thus leading to fungal cell death. Specifically, polymyxin binds to fungal cell wall synthase, blocking the enzyme activity and inhibiting the synthesis of fungal cell wall polysaccharides, which leads to the termination of fungal cell wall synthesis. This results in severe disruption of fungal cell wall synthesis and thinning of the cell wall. In addition to interfering with the synthesis of the fungal cell wall, CBZ can also interfere with the activity of enzymes related to the fungal cell wall, affecting the function of the fungal cell wall, so that the fungal cell cannot normally absorb nutrients and excrete metabolites, resulting in the inability of the fungal cell to grow and reproduce normally. CBZ can also inhibit the metabolic process of fungal cells by interfering with the enzyme activity inside the fungal cells, leading to the death of fungal cells. In addition, CBZ controls fungal diseases of *Ascomycetes* and Anamorphic fungi by preventing the growth of fungal bud tubes, attachment formation, and mycelial growth, as well as interfering with the formation of spindle bodies during mitosis of pathogenic fungi (Salunkhe et al., 2014; Zhang T. et al., 2022). It is extensively utilized globally to control a wide range of diseases in crops, fruits, and vegetables (Wang et al., 2010), including but not limited to bananas, mangoes, strawberries, oranges, pineapples, pears, cereals, sugar beets, fodder beets, rapeseed, and ornamental plants against rice blight, wheat blast, cucumber anthracnose, and canola botrytis (Liu et al., 2022). In addition, CBZ is also used in the paint, textile, and paper industries (Ebedy et al., 2022). Recently, it has also been approved for use in the leather and paint industries to improve the quality and longevity of materials (Singh et al., 2019).

Potential residue buildup in the finished product is the main issue farmers face. According to studies, CBZ has a half-life of 2 to 25 months in water under aerobic and anaerobic conditions, 3 months on turf, and 6 to 12 months in bare soil (Alvarado-Gutiérrez et al., 2017). People are growing more aware of the need of eating healthily and are also increasingly sensitive to pesticide residues in food. The discovery that CBZ is one of the main pollutants found in food has drawn a lot of attention (Shi et al., 2022). Food, contaminated water, and air are known to be the ways that CBZ enters the human body and the food chain. Nearly all of the CBZ in the dried tea leached into the brewed tea, according to Zhou et al. (2018) evaluation of the residue transfer and risk assessment of CBZ in green tea. According to Li et al. (2017), who evaluated the transfer of CBZ residues from rapeseed flowers to beekeeping products, the CBZ residues in pollen on day eighteen were 1.10 ± 0.03 mg/kg, in honey on day twenty-four were 0.032 ± 0.001 mg/kg, and in royal jelly on day twenty-two were 0.077 ± 0.002 mg/kg. A concentration of 0.01 μg/L of CBZ is detected in the surface water at the Kibuye sampling point of Lake Kivu (Houbraken et al., 2017). CBZ leftovers were detected in over 80% of African palm crop samples, samples taken close to cities, and samples taken in locations where maize crops predominated. The concentration of CBZ residues in urban areas was found to be the highest at 31 ng/L (Cabrera et al., 2023). According to Rico et al. (2022), CBZ was found in 80% of water samples taken from urban streams in the Brazilian Amazon, with concentrations as high as 214 ng/L. Because of the buildup of residues from the continuous use of CBZ, human health is today seriously threatened (Salunkhe et al., 2014). Numerous studies have shown that CBZ not only affects development, but also damages the kidneys, interferes with the body’s endocrine system, causes hematologic toxicity, forms aberrations, is toxic to reproductive tissues, leads to abnormalities in liver function, causes changes in the kinetics of microtubule assembly and disassembly, alters the stability of the meiotic spindle microtubules and the integrity of the spindle poles, and causes neuroinflammation (Prashantkumar et al., 2012; Rama et al., 2014; Alvarado-Gutiérrez et al., 2017; Long et al., 2021; Shi et al., 2022).

The following two factors can be our starting points for resolving the conflict between environmental pollution and high and consistent agricultural product yields. Pesticides with low toxicity, high efficiency, and low residue should be searched out and produced; on the other hand, essential emphasis should be paid to techniques of degradation pesticide residues (Huang et al., 2018). While CBZ can naturally deteriorate, the degrading effects of sunlight are not readily apparent (Bai et al., 2017). Hence, there is a pressing need to develop more efficient degrading techniques. Several techniques, including abiotic degradation and biodegradation, have been developed to efficiently degradation CBZ. It has been demonstrated that a few abiotic degradation processes, including photodegradation (Yogesh Kumar et al., 2022), molecular hydrogen degradation (Zhang T. et al., 2022), acoustic ozone (O_3_/US) degradation (Siddique et al., 2021), and gamma radiation (Ciarrocchi et al., 2021), are efficient in degrading CBZ. However, in addition to being harmful to the environment, the degradation of CBZ through gamma radiation, burning, electro-oxidation, microwave induction, or chemical oxidation also necessitates a large and costly infrastructure (Zhang et al., 2020). Therefore, microbial degradation—a technology that is low-cost and environmentally friendly compared to abiotic degradation methods—is attracting increasing attention.

Since it started in the 1940s, microbial degradation of bactericides has proven an efficient way to degrading complex organic pollutants into smaller, simpler molecules. For example, the majority of the pollutants are either entirely broken down into CO_2_ and H_2_O or degrading into less hazardous forms (Mishra et al., 2021b). Furthermore, microbial decomposition can withstand harsh environmental conditions and is inexpensive, making it a very viable and efficient remediation method (Mishra et al., 2021a). Numerous microbes have been shown to be efficient in degrading CBZ to date, including: *Bacillus subtilis* (Salunkhe et al., 2014), *Pseudomonas* (Alvarado-Gutiérrez et al., 2017), *Stenotrophomonas* sp. (Alvarado-Gutiérrez et al., 2017), *Rhodococcus* (Long et al., 2021), *Ralstonia* (Zhang et al., 2005), *Sphingomonas paucimobilis*, *Aeromonas hydrophila*, *Burkholderia cepacian* (Pandey et al., 2010), *Microbacterium* sp. (Zhang Y. et al., 2017), *Brevibacillus* sp. (Kanjilal et al., 2018), *Streptomyces* sp., *Rhizobium leguminosarum* (Kanjilal et al., 2018), *Klebsiella*, *Flavobacterium*, *Stenotrophomonas* (Alvarado-Gutiérrez et al., 2020), *Ralstonia* sp. (Silambarasan and Abraham, 2020), *Chryseobacterium* sp., *Aeromonas caviae* (Silambarasan and Abraham, 2020), *Enterobacter* (Cycoń et al., 2011), *Alternaria alternata and Trichoderma* sp. (Chuang et al., 2021), *Nocardioides* (Pandey et al., 2010). It was discovered that *Rhodococcus* sp. had the highest number of bacterial strains capable of degrading CBZ, and that the number of bacterial strains degrading CBZ was significantly larger than that of fungal and actinomycete strains. Such as CBZ can be effectively degraded by *Rhodococcus jialingiae* djl-6-2, *Rhodococcus qingshengii* djl-6, *Rhodococcus erythropolis* djl-11, *Rhodococcus* sp. CX-1, *Rhodococcus erythropolis* JAS13, *Rhodococcus* sp. D-1, *Rhodococcus erythropolis* CB11.

As CBZ residues receive increased attention, there has been a deeper examination of CBZ’s degradation process and mechanism (Huang et al., 2018). The primary degradation process involves the hydrolysis of CBZ to 2-aminobenzimidazole (2-AB), the predominant degradation product. It is subsequently changed into 2-hydroxybenzimidazole (2-HB), which undergoes ring cleavage to progressively degrade into CO_2_ and H_2_O (Bai et al., 2017; Long et al., 2021). According to the mechanisms of degradation that have been documented, CBZ is broken down by hydroxylation of C-N bonds in the parent molecule, which results in the formation of metabolites (Alvarado-Gutiérrez et al., 2017). Moreover, it was discovered that one of the major transformations in the degradation process of benzimidazole was aromatic hydroxylation (Shi et al., 2022). With the in-depth exploration of the degradation mechanism, there are more and more reports on CBZ degradation by functional enzymes encoded by genes (Zhang et al., 2020). including MheI (Alpha/beta fold hydrolase), CbmA (Amidase), and CYPs1A2 (Cytochrome P450 Enzyme 1A2) are the primary enzymes in charge of CBZ degradation (Shi et al., 2022; Zhang M. et al., 2022). Furthermore, degradation genes found in plasmids and chromosomes may control CBZ degradation. The hydroxylase and exo-diol dioxygenase-encoding *hdx* and *edoA* genes, as well as the aggregation of the *edoB3*, *edoB1, edoB2,* and *edoC* genes, and the aggregation of the *mno, benA,* and *catA* genes, are all implicated in the degradation of the CBZ, according to later investigations (Long et al., 2021). Much of the current discussion of carbendazim focuses on its toxicity, ecological risks, and potential risks to human health and physicochemical degradation pathways. Thus, the purpose of this review is to clarify the risks associated with CBZ residues. To describe the methods and metabolic pathways of CBZ degradation, in particular the microbial degradation pathway and to outline the metabolic pathways of the genes and enzymes that degrade CBZ and are essential to the biodegradation process. Furthermore, a number of bioremediation techniques for cleaning up soil polluted with CBZs were discussed. It is dedicated to removing CBZ residues even further and offering the theoretical foundation required for the advancement and use of the technique for utilizing microorganisms to remediate CBZ-contaminated soil.

## 2 Toxicity of carbendazim

As frequently as every 10 to 15 d, fungicides are used in agricultural production to control crop diseases. The majority of fungicides used find their way into the soil environment, where they leave behind residues that contaminate the environment (Liang et al., 2023). According to a growing body of research, fungicide residues influence soil microbial activity, microbial community structure, and function in addition to causing the emergence of microbial community resistance and the transmission of related resistance genes (Fang et al., 2016). Because of its strong toxic effects and high degree of non-degradability, CBZ has been categorized by the World Health Organization as dangerous (Ling et al., 2016). Australia, the European Union (except Portugal), and the United States have outlawed the use of CBZ; but, the United Kingdom, Portugal, and some developing countries (China, Brazil, and India) continue to permit the production and use of CBZ in various formulations (Chuang et al., 2021).

**Figure 2:**
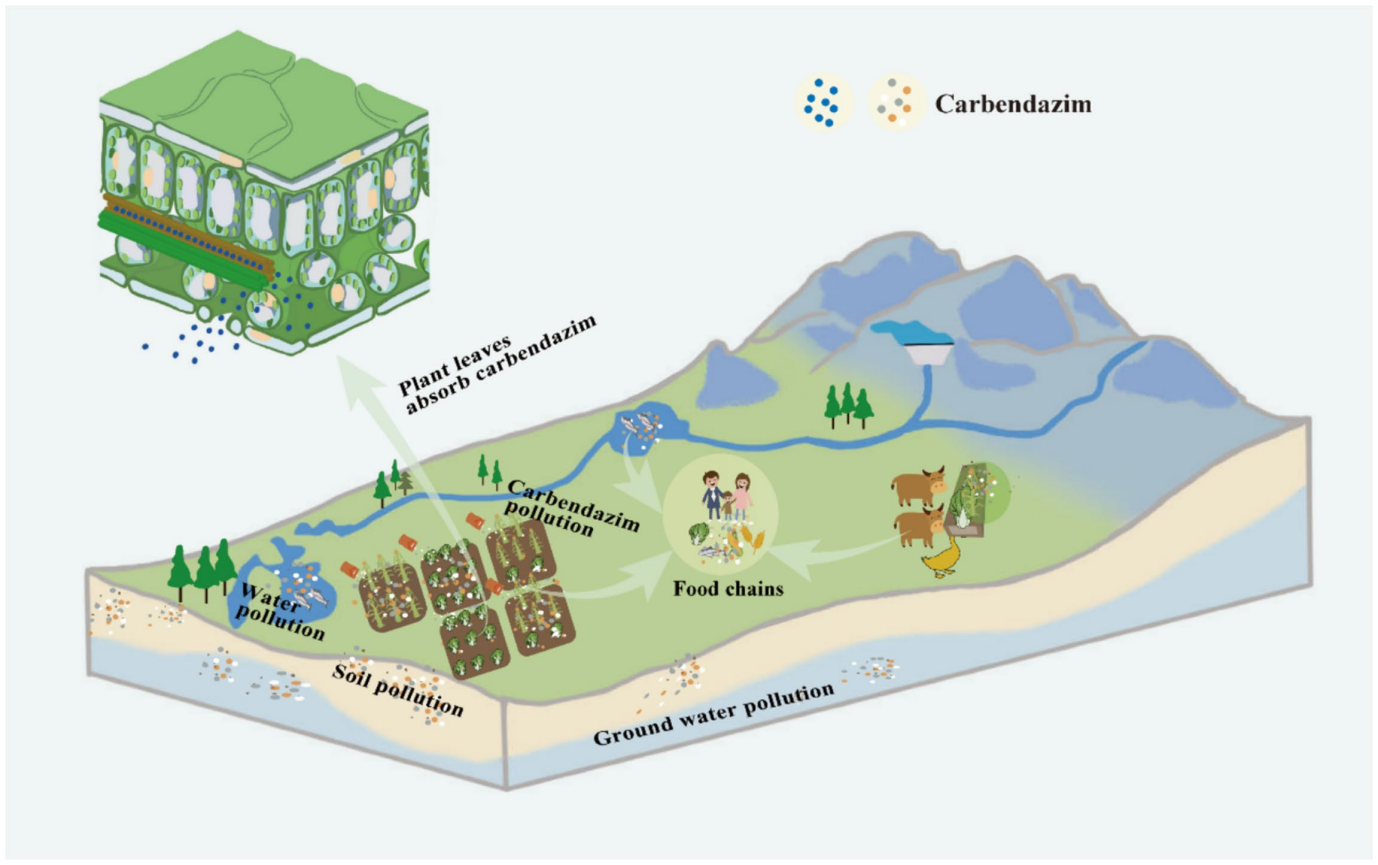
Mobility of carbendazim in the environment.

CBZ has a lengthy half-life of 6 to 12 months in bare soils because of its slow breakdown. In water, however, it dissipates rather rapidly as indicated by short half-life of about 2–25 days (Singh et al., 2016). In addition to the possible persistence of CBZ residues on leaves. Furthermore, plants have the ability to absorb CBZ through their roots, seeds, or leaves, and subsequently distribute it throughout their entire body (Silambarasan and Abraham, 2020). More dangerously, CBZ has the ability to transferred throughout the food chain, leading to bioaccumulation and biomagnification (Salunkhe et al., 2014) (Figure 2). Even CBZ has been discovered in house dust and drains (Shi et al., 2022). It has been documented that CBZ has harmful effects on amphibians, macroinvertebrates, zooplankton, and primary producers that live in freshwater, as well as people (Ling et al., 2016). Hepatocyte dysfunction, for instance, can result from CBZ-induced hepatic necrosis, hepatocyte edema, and hepatocyte degeneration (Shi et al., 2022). Significant histopathological alterations in the kidney may also result from it. These include elevated levels of blood urea nitrogen and plasma creatinine, which can cause renal dysfunction; a decrease in the kidney’s ability to eliminate excess metabolic waste products like urea and creatinine, which can cause renal vascular congestion, peritubular and periglomerular inflammatory cell infiltration, renal necrosis, and vacuolization (Sharma et al., 2022). Research has also indicated that CBZ can lead to hematological toxicity, alter hemopoiesis, have endocrine-disrupting effects, lower total platelet counts, and diminish red blood cell counts by 3–19% and white blood cell counts by 18–35% (Singh et al., 2016). Additionally, when exposed to CBZ at levels greater than 0.79 mg/L, reproductive tissues may become toxic and developmental consequences may occur (Ebedy et al., 2022). Specifically, CBZ can lead to oxidative stress on the testes and inert cells, cause testicular damage and infertility through the shedding of the germinal epithelium, affect the levels of testosterone, luteinizing hormone, follicle-stimulating hormone, inhibit testicular steroidogenesis, increase estrogen, and increase levels of androgen receptor mRNA. CBZ has also been linked to the hypothalamus-pituitary-gonadal axis being affected, the hypothalamic–pituitary-gonadal axis is impacted by CBZ, which can lead to severe estrogen-mediated lesions, changes in testicular weight, obstruction of the output ducts, shedding of germ cells, decreased spermatocyte counts and spermatozoa viability, morphologic abnormalities, failure of spermatogenesis by apoptosis of the germ cells, and infertility. Worrisomely, it has been shown that CBZ is hazardous to the fetus and can induce fetal malformation (Sharma et al., 2022).

Moreover, CBZ disrupts spindle protein meiosis, resulting in chromosomal abnormalities and aneuploidy, both of which increase the risk of cancer (Ling et al., 2016; Bai et al., 2017; Long et al., 2021). CBZ disrupts microtubule assembly and impairs chromosome segregation during cell division in fungal and mammalian cells by inhibiting both the binding of guanosine triphosphate (GTP) to microtubule proteins and microtubule polymerization through interaction with β-microtubule proteins (Rama et al., 2014), it also modifies the stability of meiotic spindle microtubules and the integrity of the spindle pole by interfering with the dynamics of microtubule construction and disassembly (Prashantkumar et al., 2012). Additionally, by activating the NF-kB signaling pathway linked to IL-1 and TNF-α cytokines, CBZ caused neuroinflammation. This resulted in time-and dose-dependent oxidative stress injury, neurobehavioral changes, and neuropathological changes, including neuronal death in various brain regions (Ebedy et al., 2022). Crucially, scientists tracked how phytotoxicity changed as CBZ biodegraded and discovered that toxicity progressively dropped as CBZ degradation (Bai et al., 2017). Thus, there has been a lot of focus in public health on the breakdown of CBZ into less dangerous molecules or its direct breakdown into CO_2_ and H_2_O.

## 3 Abiotic degradation of carbendazim

### 3.1 Overview of abiotic degradation strategies

As the general population’s worry over CBZ residues grows, more scientists are looking into the best ways to degrade CBZ. Numerous abiotic techniques have been reported to degrade CBZ, according to the literature. One of the numerous methods that contributes to CBZ degradation, for instance, is photolytic degradation (Yogesh Kumar et al., 2022). Furthermore, the inclusion of catalysts in the presence of light can efficiently degrade CBZ; Photocatalysts are widely used for the photodegradation of a wide range of pollutants, and through their use many organic, inorganic and microbiological pollutants can be converted into less harmful metabolites. One of the most often used and effective photocatalysts for the photodegradation of CBZ is titanium dioxide. Additionally, was demonstrated that when exposed to sunshine, Fe doped with TiO_2_ exhibits higher photoactivities than undoped TiO_2_. The combination of Iron (Fe) and sunlight enhances the photocatalytic activity of TiO_2_. Fe-doped TiO_2_ broke down 98% of the CBZ in direct sunlight (Kaur et al., 2016). Mungsuk et al. (2023) used particulate and sol–gel coated filters to degrade CBZ using TiO_2_ photocatalysis under sunlight, it achieved 89–91% degradation of CBZ with an optimum kinetic rate constant of 0.048 min^-1^. The type II Bi_2_S_3_/BiFeO_3_ heterojunction system is also a photocatalyst that can effectively degrade CBZ, BiFeO_3_ nanoplates and Bi_2_S_3_ nanorods are in intimate microscopic contact within the Bi_2_S_3_/BiFeO_3_ materials. The Bi_2_S_3_/BiFeO_3_ materials demonstrated superior absorption of visible light, heightened separation of charge carriers, and effective photocatalytic activity, attaining 96% degradation in just 2 hours of reaction time (Bhoi et al., 2018). Another photocatalyst, Two-dimensional (2D) gadolinium tungstate nanosheets (GW Nfs) were synthesized by Periyasamy et al. (2019). using co-precipitation. These unique structures are particularly effective because they catalyze the degradation of CBZ, a post-harvest fungicide, and other reactions. GW Nfs effectively degrades 98% of CBZ when exposed to visible light. Moreover, using electroless template synthesis, Altynbaeva et al. (2022) created a new Cu_2_O/ZnO@PET hybrid composite. The Cu_2_O/ZnO@PET composite showed increased photocatalytic activity with 98% CBZ breakdown when exposed to UV–visible light. For the first time, Wang H. et al. (2023) used Bismuth Oxyhalide (BiOCl, BiOBr, and BiOI) photocatalysts for the mineralization and degradation of CBZ. After 3 hours of exposure to a metal halide lamp at a pH of 7, the degradation efficiency reached 99%, and more than 90% of the CBZ molecules could be mineralized.

Based on physiological and genetic evidence, molecular hydrogen is thought to be a key player in controlling the degradation of CBZ in plant leaves by encouraging the synthesis of glutathione (GSH). The reasoning behind this is that two enzymes involved in the metabolism of reduced GSH are glutathione reductase (GR) and glutathione S-transferase (GST). The reduction of oxidized glutathione (GSSG) to GSH is carried out by GR, whereas the generation of GSH is stimulated by molecular hydrogen through the enhancement of γ-glutamylcysteine synthetase (γ-ECS) activity. CBZ residues have the potential to bind to GSH and create less reactive and toxic conjugates that can be transported and degraded later on, thanks to increased GST activity (Zhang T. et al., 2022). In addition, it was demonstrated that gamma radiation was useful in lowering CBZ residues in strawberries, the degradation rate according to the gamma radiation dose and the fungicide’s chemical composition (Ciarrocchi et al., 2021). Dielectric barrier discharge (DBD) plasma can cause CBZ in sealed containers to degrade either directly or indirectly. It can do this by producing a range of physical (UV and shockwave) and chemical (-OH, -O, -H radicals, O_3_, and H_2_O_2_). CBZ (0.5 ug/mL) was degraded by 89.54% under ideal circumstances (Wang J. et al., 2023). Also, by encapsulating CBZ in superabsorbent hydrogels (SHs), CBZ’s dissipation rate increased dramatically by 34.2–54.1%, reducing its persistence in the soil matrix (Yang et al., 2018). And in order to treat various forms of CBZ-polluted wastewater, aluminum carbide nanosheets (AlC_3_NS) were employed as an effective adsorbent. It was shown that the adsorption energy of CBZ through-C=O groups interacting with Al atoms of AlC_3_NS was around 30.14 kcal/mol (Kadhim et al., 2022). Besides, the fungicide CBZ is degraded by the combined application of ozone and ultrasound (O3/US), which reduces the average CBZ residue in fruits and vegetables by 72% (Siddique et al., 2021). Yogesh Kumar et al. (2022) synthesized gadolinium sesquisulfide anchored with nitrogen-doped reduced graphene oxide (Gd_2_S_3_/NRGO) by a simple microwave-assisted method. It was found that Gd_2_S_3_/NRGO could effectively degrade CBZ, and 94% of CBZ was degraded within 90 min in the presence of Gd_2_S_3_/NRGO.

In summary, the degradation of CBZ by these abiotic degradation methods mainly focuses on the application of photocatalysts, and a few of them use some physical or chemical means to degrade CBZ. The non-biological degradation method is mainly characterised by high degradation efficiency, but there are some disadvantages of this method, such as the slow degradation rate and the long period required for natural photolytic degradation; and the degradation of CBZ by gamma radiation is not environmentally friendly; The study of CBZ degradation by particulate and sol–gel coated filters, Bi_2_S_3_/BiFeO_3_ heterojunction materials and novel composite materials requires a large amount of costly infrastructure, which is expensive; Photocatalyst degradation of CBZ is inexpensive, green and environmentally friendly, but the production process of photocatalysts is cumbersome and takes a long time; therefore, the development of microbial degradation of CBZ, which is cheaper, greener and easier to implement, is gradually becoming a hot research topic.

### 3.2 Abiotic degradation pathways

**Figure 3:**
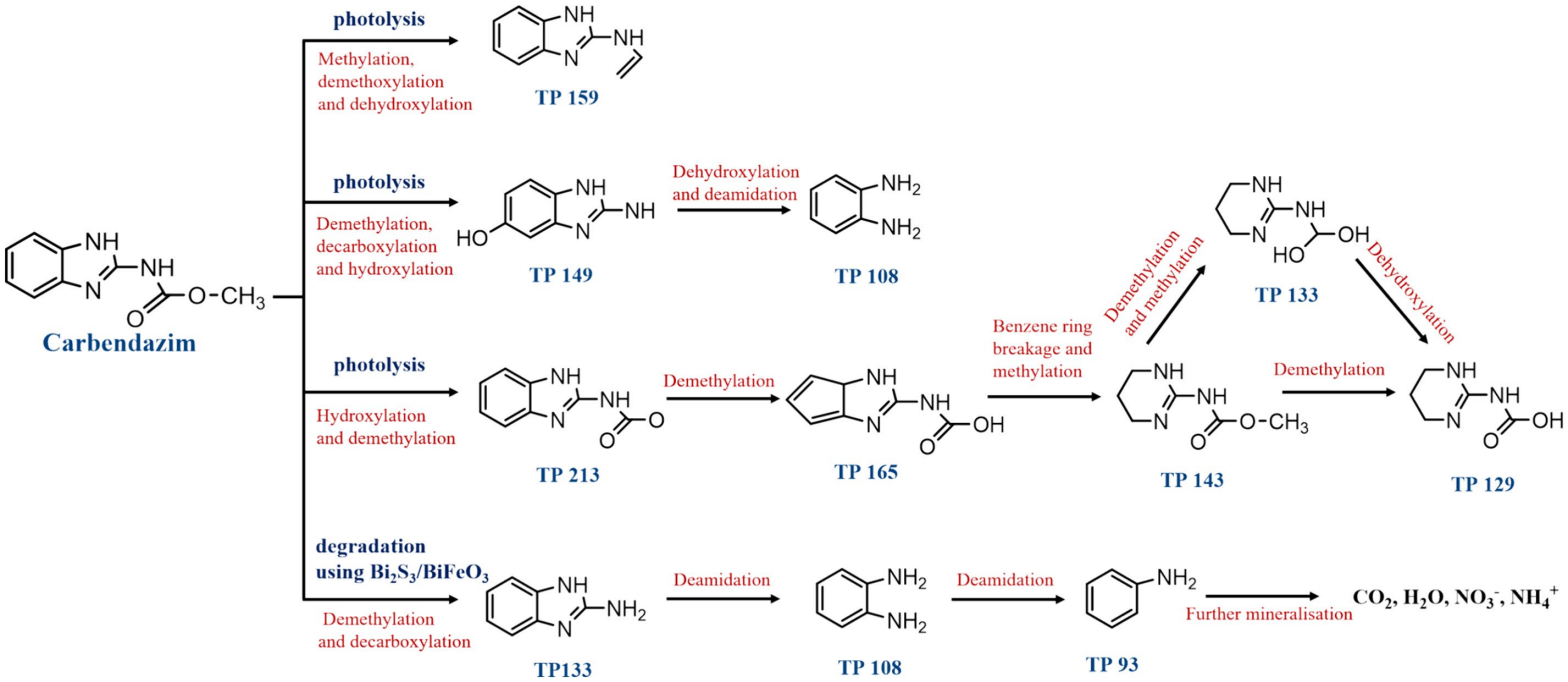
Degradation pathways of carbendazim by non-biodegradable.

The spontaneous degradation of CBZ in soil occurs via three different metabolic routes (Figure 3). The first route involves producing TP159 by methylating, deoxymethylation, and dehydroxylating CBZ. The second pathway of degradation involves the production of TP 149 through the demethylation, decarboxylation, and hydroxylation of CBZ. Alternatively, TP 159 can be formed through deoxymethylation and hydroxylation. Deoxymethylation and deamidation resulted in the production of TP 108 (1,2-diaminobenzene), and comparatively high quantities of TP 149 and TP 159 were found, indicating that these intermediates are the main metabolites. The third degradation process produces TP 213 by hydroxylating and demethylating CBZ. After the benzene ring is split, TP 165, TP 143, TP 133, and TP 129 are formed as a consequence of a number of processes, including deoxymethylation, demethylation, and methylation (Huang et al., 2020). Moreover, employing Bi_2_S_3_/BiFeO_3_ heterojunction materials as photocatalysts, a fourth degradation route has been discovered for the effective photocatalytic breakdown of CBZ fungicide under visible light irradiation. When CBZ broke down on the surface of the Bi_2_S_3_/BiFeO_3_ catalysts, aliphatic organic intermediates were released and the intermediates TP = 133 and TP = 108 formed. Following the release of NH_3_, the intermediate TP = 108 produces aniline (intermediate TP = 93). TP = 93 takes over as the primary intermediate in the photocatalytic mineralization of CBZ after 60 min. CO_2_, H_2_O, NH_4_^+^, and NO_3_^−^are produced when the generated intermediate is further mineralized on the catalyst surface (Bhoi et al., 2018). Numerous studies have been conducted on the abiotic degradation of CBZ, Nevertheless, the majority of them solely concentrate on creating novel degrading techniques and raising the effectiveness of degradation. Few comprehensive studies have been conducted on the mechanism and degradation pathway of CBZ; therefore, it is important to continue researching the degradation pathway in order to degrade the CBZ more quickly and effectively. The investigation of the CBZ degradation process needs more attention in the future.

## 4 Microbial degradation of carbendazim

### 4.1 Potential microorganisms for degradation of carbendazim

Multiple methods have been developed to degrade CBZ in response to growing environmental awareness. Nevertheless, it was discovered that photolysis was weak for CBZ degradation (Bai et al., 2017), and that certain abiotic degradation techniques typically call for the use of specific catalysts or high doses of radiation and ozone, which could present new hazards to food safety (Wang J. et al., 2023). Consequently, the development of an environmentally acceptable and efficient approach for CBZ degradation becomes very vital. More and more efforts are being made to use microbial degradation for the degradation of CBZ because, as scientific research technology advances, researchers have discovered that microbial degradation is crucial to the biodegradation of pesticides, which can transform pesticides into forms that are safe for the environment and non-toxic (Ling et al., 2016).

**Figure 4:**
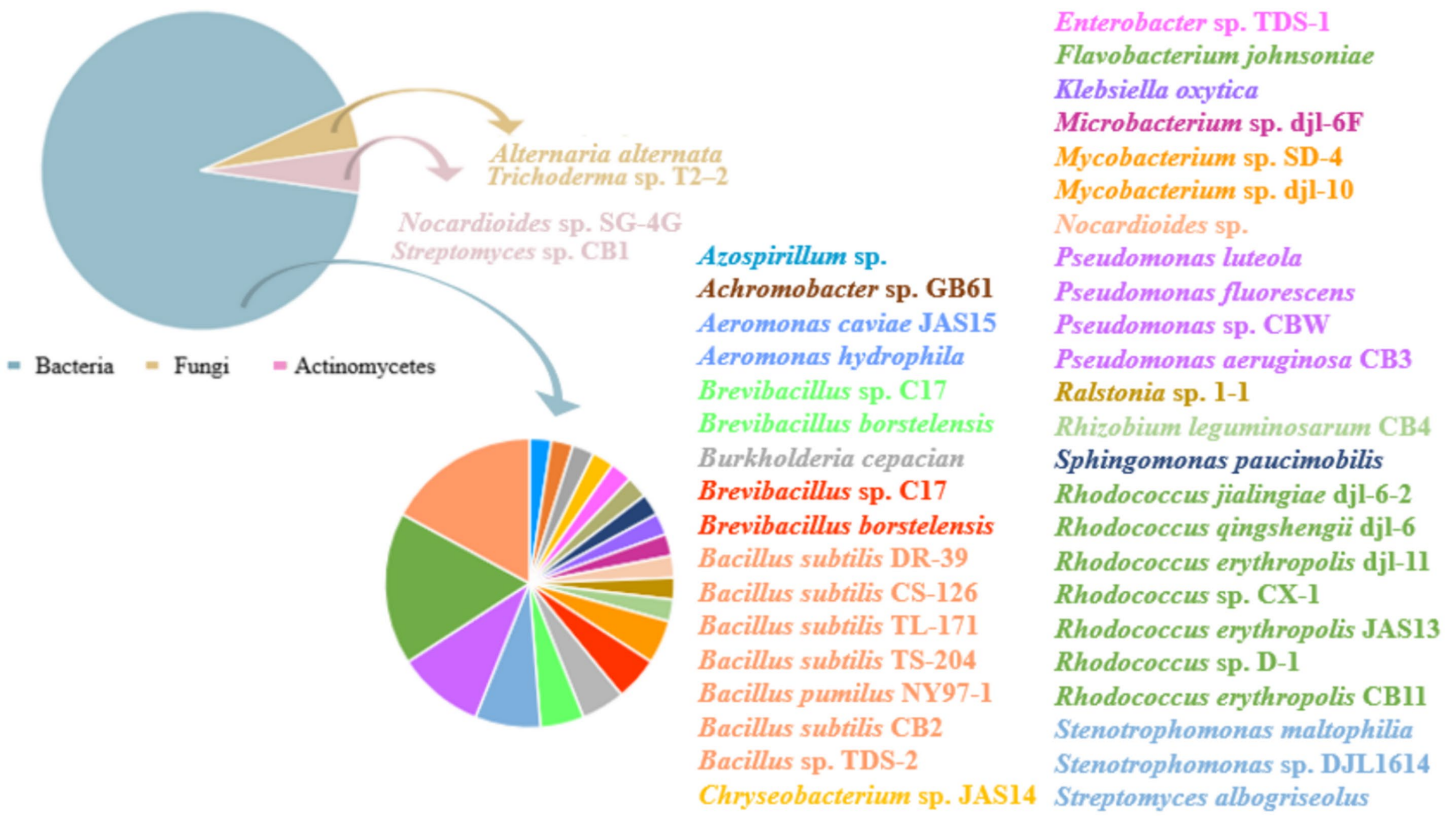
Carbendazim degrading strains. Different color block regions in the pie chart indicate the proportion of this species to found strains.

Numerous microbial strains have been discovered that degrade CBZ at this time. In general, bacteria are more important in CBZ biodegradation than fungi and actinomycetes are in the dissipation of CBZ in soil (Figure 4) (Yarden et al., 1990). A multitude of *Rhodococcus* sp. strains were discovered to be efficient in degrading CBZ, as indicated in Table 1. Among these strains were *Rhodococcus erythropolis* JAS13 (Silambarasan and Abraham, 2020), *Rhodococcus* CX-1 (Long et al., 2021), *Rhodococcus jialingensis* djl-6-2 (Wang et al., 2010), *Rhodococcus erythropolis* djl-11 (Zhang et al., 2013), *Rhodococcus* sp. D-1 (Bai et al., 2017), *Rhodococcus qingshengii* djl-6 (Jing-Liang et al., 2006; Chuang et al., 2021), The ability of these strains to degrade CBZ is demonstrated in Table 1. Additionally, it has been discovered that certain bacteria from different genera may effectively degrade CBZ. For example, *Achromobacter* sp. GB61 can degrade CBZ quite effectively. Following treatment with *Achromobacter* sp. GB61, the half-life of CBZ was 8.45 d, as opposed to 63 d in the absence of this strain. Furthermore, the breakdown intermediates’ metabolites were found to be environmentally acceptable and non-toxic (Ling et al., 2016). The degradation of CBZ by four strains of *Bacillus subtilis* (DR-39, CS-126, TL-171 and TS-204) was examined by Salunkhe et al. (2014). Thompson Seedless was sprayed with 1.0 g/L of CBZ, and after 25 d, the residual on control grapes was 0.44 mg/kg; however, the residue on grapes treated with the four *Bacillus subtilis* strains was just 0.02 mg/kg. In treated grapes, degradation kinetics revealed a reduced half-life, ranging from 3.1 to 5.2 d, as opposed to 8.8 d in the control group. Moreover, when concentrations increased (10 to 300 mg/L), *Bacillus pumilus* NY97-1’s CBZ degradation ranged from 42.44 to 90.07% (Zhang et al., 2009). In M9 Minimal Medium supplemented with 250 mg/L CBZ, Song and Hwang (2023) demonstrated that the *Bacillus velezensis* HY-3479 strain could digest CBZ. Its optimal degradation efficiency was achieved 48 h after the addition of 12.5 mM NH_4_NO_3_.

**Table 1:** Carbendazim-degrading strains and degradation efficiency.

Strain	Environment	Time	Concentration	Degradation
*Bacillus pumilus* NY97-1	MSM	24 h	300 mg/L	Degradation rate of 90.07%
*Brevibacillus panacihumi* C17	MSM	36 h	100 mg/L	Degradation rate of 87.25%
*Bacillus velezensis* HY-3479	M9 minimal medium	48 h	250 mg/L	Degradation rate of 76.99%
*Rhodococcus jialingensis* djl-6-2	MSM	60 h	100 mg/L	Degradation rate of 94%
*Rhodococcus* sp. D-1	MSM	3 d	200 mg/L	Degradation rate of 98.2%
*Streptomyces* CB1	MSM	14 d	1,000 mg/L	Degradation rate of 91.65%
*Pseudomonas aeruginosa* CB3	MSM	14 d	1,000 mg/L	Degradation rate of 87.35%
*Bacillus subtilis* CB2	MSM	14 d	1,000 mg/L	Degradation rate of 81.85%
*Rhodococcus erythropolis* JAS13	MSM	-	150 mg/L	Degradation rate of 100%
*Rhizobium leguminosum* CB4	MSM	14 d	1,000 mg/L	Degradation rate of 76.54%
*Brevibacillus borstelensis* and *Streptomyces albogriseolus*	MSM	12 h	30 ug/mL	Degradation rate of 97%
*Mycobacterium* sp. SD-4	MSM	72 h	50 mg/L	Average degradation rate of 0.63 mg/L/h
*Rhodococcus erythropolis* djl-11	MSM	–	1,000 mg/L	Average degradation rate of 333.33 mg/L/d
*Rhodococcus* sp. CX-1	MSM	5 h	50 mg/L	Average degradation rate of 9.90 mg/L/h
*Rhodococcus qingshengii* djl-6	M9 minimal medium	36 h	100 mg/L	Average degradation rate of 55.56 mg/L/d
*Pseudomonas* sp. CBW	Mineral salts medium	3 d	10.0 mg/L	Degradation rate of 99.1%
*Ralstonia* sp. 1–1	Addition of yeast extract in liquid MSM medium	24 d	500 mg/L	Degradation rate of 95.96%
*Bacillus subtilis* DR-39, CS-126, TL-171, TS-204	Thompson Seedless	25 d	1.0 g/L	Degradation rate of 95.45%
*Chryseobacterium* sp. JAS14	Soil	9 d	200 mg/L	Degradation rate constant of 27.30 d-^1^
*Aeromonas caviae* JAS15	Soil	9 d	200 mg/L	Degradation rate constant of 23.87 d^−1^
*Trichoderma* sp. T2-2	Sterilized soil	6 d	50 mg/L	Degradation rate of 100%

Besides, CBZ was demonstrated that *Brevibacillus panacihumi* C17 also efficiently degraded CBZ by 87.25% at a concentration of 100 mg/L over an incubation period of 36 h, pH 7.0, and 180 rpm/min (Kanjilal et al., 2018). After 3 d of incubation at concentrations of 1.0 and 10.0 mg/L, respectively, *Pseudomonas* sp. CBW decomposed approximately 87.1 and 99.1% of CBZ in MSM (Fang et al., 2010). *Streptomyces* CB1, *Pseudomonas aeruginosa* CB3, *Bacillus subtilis* CB2, and *Rhizobium leguminosum* CB4 decompose 91.65, 87.35, 81.85, and 76.54% of CBZ, respectively, throughout the degradation process (Singh et al., 2019). And the degradation rate constants were 27.30 d^−1^ and 23.87 d^−1^, respectively, after *Chryseobacterium* sp. JAS14 and *Aeromonas caviae* JAS1 were introduced into soil without additional nutrients (Silambarasan and Abraham, 2020). *Ralstonia* sp. 1-1’s degradation rate constants in MSM supplemented with yeast extract (150 mg/L) and CBZ (500 mg/L) revealed a degradation rate of 19.16 and 95.96%, respectively, within 24 d (Zhang et al., 2005). Using CBZ as the only source of carbon and nitrogen for development, *Mycobacterium* sp. SD-4 was able to degrade 50 mg/L CBZ at an average degradation rate of 0.63 mg/L/h (Zhang Y. et al., 2017). In addition, *Stenotrophomonas* sp. DJL1614 also is a kind of bacteria that breaks down CBZ pesticide residues, according to a Chinese patent (Alvarado-Gutiérrez et al., 2017). And LC–MS analysis showed that the combined degradation of CBZ by *Streptomyces albogriseolus* and *Brevibacillus borstelensis* decreased the concentration of CBZ from 30 ug/mL to 0.86 ug/mL in 12 h with a degradation rate of 97%, and to 0.60 ug/mL in 20 h with a degradation rate of 98% (Arya and Sharma, 2015). CBZ is also broken down by *Klebsiella oxytica*, *Flavobacterium johnsoniae*, and *Stenotrophomonas maltophilia*. All three bacteria have been shown to carry genes encoding the mheI, which is implicated in CBZ degradation (Alvarado-Gutiérrez et al., 2020). With a high conversion rate and no need for cofactors, the MheI-6F protein purified from the CBZ-degrading strain *Microbacterium* sp. djl-6F catalyzed the direct hydrolysis of CBZ to 2-aminobenzimidazole (2-AB) (Lei et al., 2017). *Bacillus* sp. TDS-2 and *Enterobacte*r sp. TDS-1 strains may also be able to convert CBZ to 2-AB, which could be used for bioremediation of CBZ-contaminated soil (Cycoń et al., 2011). And six d after being inoculated with *Trichoderma* sp. T2-2, CBZ in sterile soil was fully destroyed (Liansheng and Fei, 2009).

To sum up, there are a lot of microbial species that can break down CBZ. However, different strains have varying capacities to break down CBZ. This is because different strains have different functional enzymes, which causes differences in the strains’ ability to break down CBZ. On the other hand, different culture conditions can affect the growth of microorganisms, which can affect the efficiency of the strains’ CBZ degradation. The ideal growth conditions for various microorganisms vary depending on factors like temperature, pH, concentration of CBZ, extra sources of carbon and nitrogen, duration of incubation, etc. The ideal growth temperature for some bacteria is 37°C, whereas for others it is 30°C. Elevations or decreases in temperature will impact the development of bacterial strains. In addition, some microorganisms can use the nutrients in the soil for their growth, but some microorganisms cannot grow well in the soil environment, so in the work of soil microbial remediation, it is not only necessary to screen strains with the ability of efficiently degrading CBZ, but also need this strain to have the ability to grow well in the soil environment. For example, it was found that strains *Achromobacter* sp. GB61, *Rhodococcus qingshengii* djl-6 and *Actinomucor elegans* LBM 239 had good bioremediation effects on CBZ-contaminated soils under different conditions, which can be used for bioremediation of CBZ-contaminated environment.

### 4.2 Pathways of microbial degradation of carbendazim

**Figure 5:**
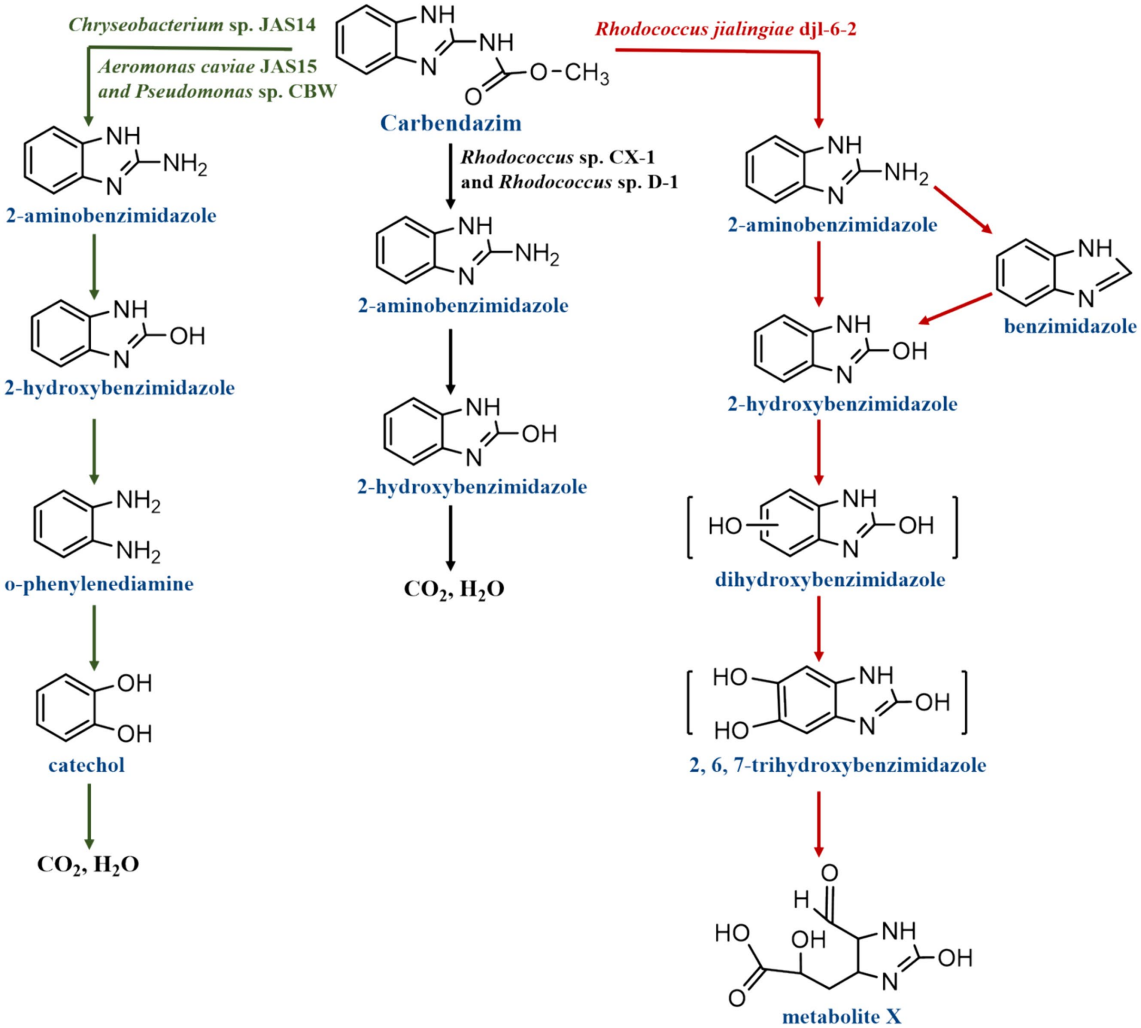
Degradation pathways of carbendazim by microbial strains.

While numerous strains have been employed for CBZ degradation, only a small number of strains have been suggested for their particular degradation pathways. Only *Rhodococcus* sp. CX-1, *Rhodococcus* sp. D-1, *Chryseobacterium* sp. JAS14, *Aeromonas caviae* JAS15, *Pseudomonas* sp. CBW and *Rhodococcus jialingiae* djl-6-2 now describe their metabolic pathway for degrading CBZ, as illustrated in Figure 5. After analyzing the molecular weight and structural formula of CBZ intermediate metabolites using HPLC-MS/MS, the following was inferred about the biodegradation process of CBZ by the strains of *Rhodococcus* sp. D-1 and CX-1: First, 2-AB was produced by hydrolyzing CBZ. Next, 2-hydroxybenzimidazole (2-HB) was produced, and finally, 2-HB was progressively broken down into CO_2_ and H_2_O (Bai et al., 2017; Long et al., 2021). In addition, *Pseudomonas* sp. CBW, *Chryseobacterium* sp. JAS14 and *Aeromonas caviae* JAS15 biodegraded CBZ by the following pathway: After converting CBZ to 2-AB, 2-HB was quickly converted. 2-HB was then further transformed by ring cleavage to o-phenylenediamine and catechol, and ultimately even to CO_2_ and H_2_O (Fang et al., 2010; Silambarasan and Abraham, 2020). The first step in *Rhodococcus jialingiae* djl-6-2’s degradation of CBZ is the hydrolysis of methyl carbonate’s side chain, which yields 2-AB or benzimidazole (BZ). It was discovered that NH_4_NO_3_ inhibits the conversion of 2-AB during this process. After then, both are transformed to 2-HB. Ring cleavage of 2-HB further opens the benzene ring to catechol (BZ) (Wang et al., 2010).

From the degradation pathway of CBZ by the six strains of bacteria, it was found that the mineralization of CBZ occurs through the hydroxylation of C-N bonds in the parent molecule to form metabolites. The CBZ degradation pathways of three strains, *Pseudomonas* sp. CBW, *Chryseobacterium* sp. JAS14 and *Aeromonas caviae* JAS15 were more clearly defined compared to the CBZ degradation pathways of *Rhodococcus* sp. D-1 and CX-1: CBZ undergoes demethylation and decarboxylation to form 2-AB, then hydroxylation to form 2-HB, after which it undergoes dehydroxylation to form o-phenylenediamine, which undergoes hydroxylation to form catechol, which is finally mineralized to CO_2_ and H_2_O. In contrast, *Rhodococcus* sp. D-1 and CX-1 were insufficient for the intermediate steps of the CBZ degradation pathway, and only CBZ was subjected to demethylation and decarboxylation to generate 2-AB, then hydroxylation to generate 2-HB, and finally 2-HB was mineralized to CO_2_ and H_2_O. In addition, a new degradation process was identified in the degradation pathway of CBZ by *Rhodococcus jialingiae* djl-6-2, whereby 2-AB can be degraded to benzimidazole and then hydroxylated to produce 2-HB. Overall, smaller molecule degradation products have yet to be discovered and further additions to the CBZ degradation pathway are still necessary.

### 4.3 Functional enzymes/genes involved in carbendazim degradation

Researchers have been studying the degradation process of CBZ more and more depth as a result of the low toxicity, efficiency, and direct degradation of pesticides by microbial enzymes that have attracted attention (Huang et al., 2018). Various enzymes have been shown to be able to achieve the degrading reaction of any organic contaminant. This is because, by catalyzing the creation of new proteins, microbial enzymes might decrease the toxicity and duration of these substances while hastening the degradation of organic contaminants (Zhang et al., 2020). Such as organic contaminants of all kinds are frequently degraded by enzymes such as ligninases, hydroxylases, cytochrome P450, hydrolases, and oxidases (Ciarrocchi et al., 2021).

**Figure 6:**
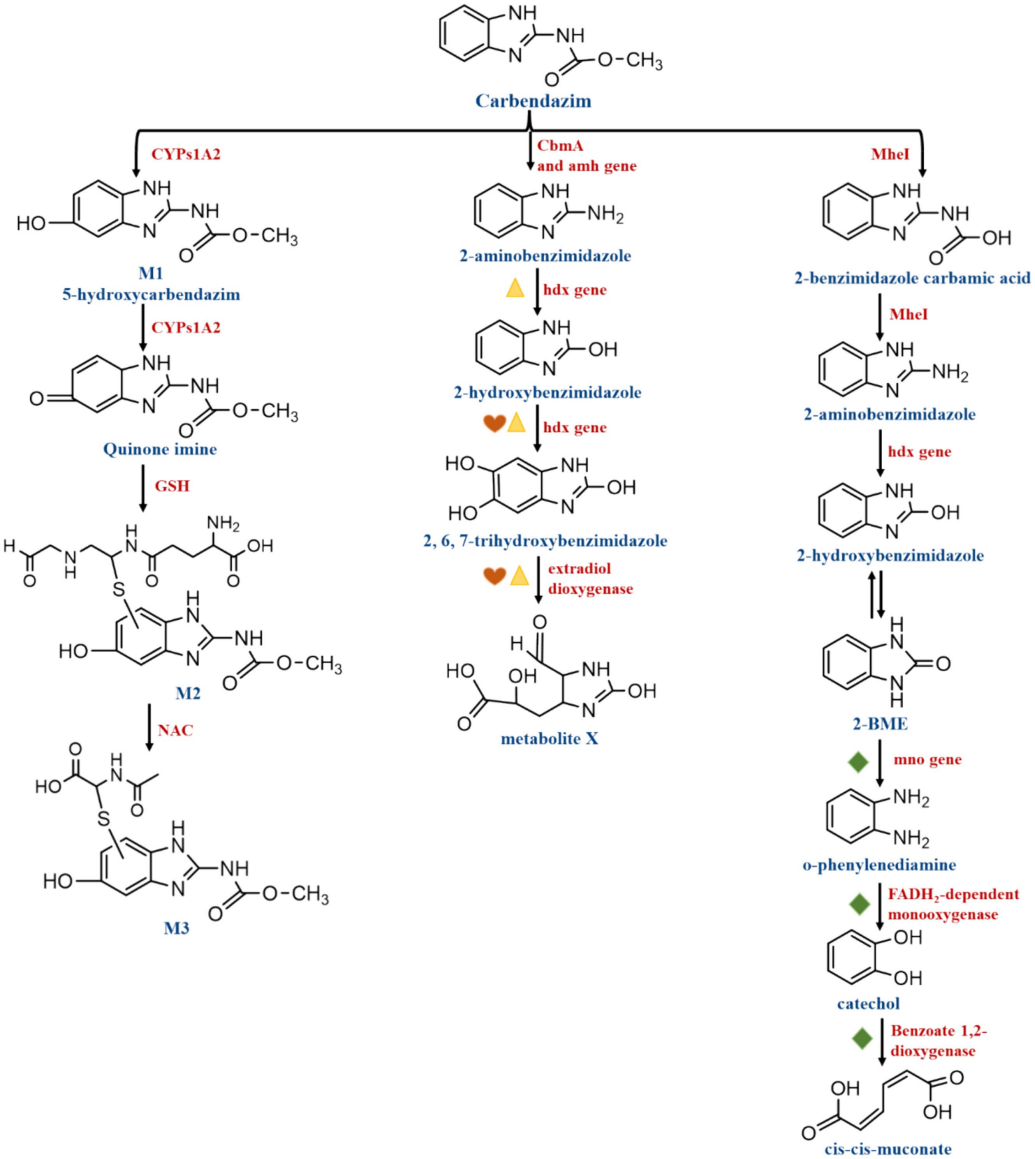
Carbendazim degradation pathways involving functional enzymes and genes. 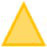 Indicate *hdx* and *edo*A genes were clustered, 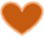 indicate *edo*B3, *edo*B1, *edo*B2, and *edo*C genes were clustered, 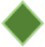 indicate *mno*, *ben* A, and *cat* A genes were clustered.

According to Zhang M. et al. (2022), the primary cause of the breaking of the CBZ ester bond to provide methanol and 2-benzimidazole carbamic acid is the CBZ hydrolase. Such as, MheI and CbmA were individually classified as esterase and amidase according to different catalytic mechanisms. Based on the conserved signature GXSXXG motif and the shared catalytic site Ser-His, MheI was identified as a member of the Abhydrolase superfamily. MheI hydrolyzed methyl salicylate, α-naphthyl acetate, and p-nitrophenyl acetate. The esterase MheI was in charge of cleaving the CBZ ester bond to produce methanol and 2-benzimidazole carbamic acid. The latter was unstable since it included both an amino and a carboxyl group that may separate spontaneously to result in 2-AB (Figure 6). With only one amino acid change at position 150, the *mheI* gene is highly conserved (>99% identity) across several CBZ degradation strains (Zhang M. et al., 2022). *mheI* was discovered to be present in the following strains: *Rhodococcus* sp. CX-1 (Long et al., 2021), *Microbacterium* sp. djl-6F (Fang et al., 2010), *Mycobacterium* sp. SD-4 (Zhang Y. et al., 2017), *Mycobacterium* sp. djl-10 (Zhang J. et al., 2017), *Nocardioides* sp. SG-4G (Pandey et al., 2010), *Rhodococcus qingshengii* djl-6 (Zhang et al., 2005; Chuang et al., 2021), *Rhodococcus erythropolis* djl-11 (Zhang et al., 2013). Furthermore, CbmA is an amidase signature superfamily enzyme containing a highly conserved catalytic triad Ser-Ser-Lys. it might have catalytic activity for compounds with the secondary aromatic amine and the N atom attached to relatively simple structures, such as acetic acid, propionic acid, or formate. It was shown to be highly conserved in *Rhodococcus* sp., hydrolyzing p-nitroacetanilide, and it was classified as a member of the amidase superfamily with catalytic site residues Lys82, Ser187, and Ser181. The amide bond of CBZ was broken by CbmA, resulting in the formation of 2-AB and methyl formate (Zhang M. et al., 2022) (Figure 6). It has also been demonstrated that the primary enzyme in the metabolism of CBZ is CYP1A2, one of the Cytochrome P450 enzymes (CYP450). CYP450 participates in the metabolism of many substances, including endogenous substances, exogenous substances and drugs. CYP450 has been found to be effective in degrading a variety of fungicides and herbicides, among others. For example, CYP90D5 one of the CYP450, promotes the degradation of isoproturon (IPU) and acetochlor (ACT) in rice tissues and grains (Su et al., 2023). CYP1A2 was found to be effective in degrading CBZ. It is most likely hydroxylated by CYP1A2 to M1 (5-hydroxycarbendazim), which is then oxidized by CYP1A2 enzymes to a quinone-imine intermediate. The resulting electrophilic species then interacted with glutathione (GSH) and N-acetylcysteine (NAC). As the most important bio-thiol, GSH, an excellent soft nucleophile, can readily react with and degrade the contaminant, a soft electrophile, with or without the assistance of glutathione transferases. In other words, quinone-imine forms M2 (glutathione conjugate) by binding to GSH, and M2 forms M3 (N-acetyl cysteine conjugate) by binding to NAC (Shi et al., 2022) (Figure 6).

When it comes to the degradation of organic compounds of genes, bacterial plasmids are crucial. In microbial strains, fungicide degradation genes are found on chromosomes, transposons, or, most frequently, plasmids (Dionisio et al., 2019). The genome of strain *Rhodococcus* sp. CX-1 was found to have 7,651 predicted genes, of which the *amh* genes located on plasmids 2_orf0354, 2_orf0356, and chr_orf9440 were determined to be crucial in the conversion of CBZ to 2-AB. Plasmids chr_orf2214 and 2_orf0061, which carry *hdx* genes, are responsible for the degradation of 2-AB to 2-HB and then 2-HB to 2, 6, 7-trihydroxybenzimidazole (2,6,7-HBM). Extradiol dioxygenase in plasmid 2_orf0059 and plasmid chr_orf2213 is responsible for the degradation step of 2,6,7-HBM to X1(C7H8N2O5), which is essential for the cleavage of the benzene ring in CBZ. In addition, it was found that the *mno* gene in plasmid chr_orf8524 was involved in the imidazole ring cleavage reaction for the degradation of 2-benzimidazolone (2-BME) to o-phenylenediamine, which could be converted to catechol by the FADH_2_-encoded FADH_2_-dependent monooxygenase encoded by plasmid chr_orf85527. Catechol was considered to be cleaved by Benzoate 1,2-dioxygenase. Dependent monooxygenase encoded by plasmid chr_orf8527 to catechol. Catechol cleavage can be achieved by Benzoate 1,2-dioxygenase encoded by chr_orf8517, and catechol is considered to be a common intermediate in the degradation of aromatic compounds. The enzymes hydroxylase and estradiol dioxygenase, encoded by the genes *hdx* and *edoA*, were found to be clustered. These genes participate in the degradation processes of 2-AB to 2-HB, then 2-HB to 2,6,7-HBM and 2,6,7-HBM to X1 (C_7_H_8_N_2_O_5_). The *edoB3*, *edoB1*, *edoB2*, and *edoC* genes were also clustered and may be involved in the degradation of 2-HB to 2,6,7-HBM, and then 2,6,7-HBM to X1 (C_7_H_8_N_2_O_5_). In the degradation of 2-BME to o-phenylenediamine, o-phenylenediamine to catechol, and catechol to cis-cis-muconate, The genes *mno*, *ben A*, and *cat A* were clustered to participate in the degradation process described above (Long et al., 2021) (Figure 6).

## 5 Bioremediation of carbendazim-contaminated environments

In modern agriculture, CBZ is widely used to keep pests out of crops, but if sprayed improperly, it can have detrimental effects on the environment and living beings (Wei et al., 2023). Because it kills or inhibits pathogenic fungi and sensitive bacteria in the soil, and because the remains of dead microorganisms can be used as a substrate to promote the growth of other resistant microorganisms, cause bacteria to become resistant to CBZ, and change the overall makeup of the bacterial community (Tarla et al., 2020). CBZ residues have the ability to alter the functional and structural diversity of soil microbial communities through both direct and indirect effects (Zhou et al., 2023). Additionally, they have the ability to significantly lower soil nitrification, ammonification, and dehydrogenase activities (Zhang M. et al., 2022).

Significantly, the stability and productivity of agroecosystems are primarily determined by soil microbial populations and related activities (Van Der Heijden et al., 2008). The removal of CBZ residues from agricultural soils has thus emerged as a pressing issue. Degradation of CBZ has been demonstrated to be an environmentally beneficial option through sustainable bioremediation of CBZ-contaminated soils. The term “bioremediation” refers to a biodegradation process that breaks down organic chemicals (such as pesticides, biocides, and other natural contaminants) contaminated by using microorganisms’ metabolic capabilities. In addition, bioremediation has drawn a lot of interest as a widely used, affordable, effective, and ecologically friendly method of cleaning up contaminated areas (Kour et al., 2021). It is commonly known that a variety of microorganisms, including bacteria, fungi, and algae, may efficiently metabolize pesticides or alter their chemical structures, accelerating their breakdown and cleaning up contaminated environments. As a result, an increasing number of people are attempting to bioremediate CBZ-contaminated environments using microbial strains or consortia (Giri et al., 2021; Sarker et al., 2021). For instance, strain *Achromobacter* sp. GB61 played a significant role in the bioremediation of CBZ-contaminated habitats and was successful in bioremediating CBZ-contaminated soil under various conditions. It also contributed to the large-scale degradation of CBZ (Ling et al., 2016). The inoculation of strain *Rhodococcus qingshengii* djl-6 into the soil was found to be beneficial for the remediation of CBZ-contaminated soil, as more than 93% of the CBZ was eliminated from the soil after 14 d of incubation, according to the researchers. It was also discovered that members of the soil microbial community, particularly soil bacteria, may benefit from the intermediate metabolites of strain djl-6’s CBZ breakdown. Thus, it also encourages the formation of additional potential CBZ-degrading microorganisms from the local microbial community in the soil (Chuang et al., 2021). Moreover, other *Rhodococcus* sp. strains that degrade CBZ also have good potential for bioremediation. One such strain is *Rhodococcus jialingiae* djl-6-2, which was inoculated into CBZ-contaminated soil. Regardless of whether the soil had been sterilized or not, the degradation rate of CBZ was higher than that of the uninoculated soil, indicating that strain djl-6-2 has a good bioremediation effect on the CBZ-contaminated soil. This suggests that on CBZ-contaminated soil, strain djl-6-2 has a good bioremediation impact. This is because *Rhodococcus* sp. exhibits a remarkable capacity to metabolize chemicals that are challenging to degrade, including pesticide derivatives that include amino and nitro, haloaromatics, aromatic compounds, and PAHs (Wang et al., 2010). Using *Actinomucor elegans* LBM 239 in combination with biostimulation proved to be a good treatment for CBZ elimination, detoxification, and soil fertility, according to Baumann et al. (2024).

Chen et al. (2021) evaluated the effectiveness of the CBZ-degrading bacterium *Streptococcus densiflorus* WJD-55 and its safety in shed tomato soils. It was found that dense *Streptomyces* sp. WJD-55 could effectively play the role of degrading CBZ and rapidly reducing the residual CBZ content in the soil. The initial soil CBZ mass concentration was about 42 mg/kg, and the use rate of the fungicide was 0.1%. On the 10th day after the treatment, the mass concentration of CBZ in the soil was 9.27 mg/kg, which decreased by 78%, while the mass concentration of CBZ in the control soil was 30.2 mg/kg, which declined by 29%. In addition, it was found that dense *Streptococcus* sp. WJD-55 could stably colonize the soil and could improve the fungal microbial diversity of contaminated soil, with no adverse effects on the soil’s physicochemical properties and the growth of tomato crops. Tian and Chen (2009) used corn stover powder as raw material to produce *Xylomycetes* T2-2 bioremediation agent through solid fermentation. The soil was artificially inoculated with the T2-2 bioremediation agent at an inoculum level of 107 cfu/g of dry soil and a CBZ content of 0.1 mg/g of dry soil for the remediation of sterilized and natural soils. It was found that the inoculation of CBZ in sterilized soil was completely degraded in 6 d, whereas the complete degradation of CBZ in natural soil was shortened to 4 d. This suggests that straw meal acts as a co-metabolizing substrate and promotes co-metabolizing degradation of T2-2 and Indigenous microorganisms. In addition, strain T2-2 was found to degrade CBZ in the soil and control plant diseases. According to Xiao et al. (2013), native soil microbes, CBZ-degrading strains, and *Sedum alfredii* worked together to remove CBZ from polluted soil. Sedum alfredii changed the community structure of the soil, boosted microbial activity, and enhanced microbial diversity. Remarkably, rather than coming from direct plant absorption, CBZ dissipation is the outcome of encouraging biodegradation. Furthermore, *Sedum alfredii* were demonstrated superior efficacy in the biodegradation of CBZ in conjunction with *Flavobacterium* and *Pseudomonas* sp. These strains enhanced microbial activity, enriched microbial diversity, and modified the structure of microbial communities, all of which contributed to the biodegradation of CBZ in the soil. To sum up, the landscape and scope of environmental remediation are being dramatically altered by these bioremediation technologies in the direction of sustainable bioremediation.

## 6 Conclusion

Fungicides are indispensable for sustainable crop production. However, as a commonly used fungicide, carbendazim, while promoting production, also caused severe fungicide contamination. Carbendazim can enter the human body and food chain through food, contaminated air and water, posing a serious threat to biological health and ecological environment. Therefore, the degradation of carbendazim residues is urgent. Studies have shown that many microbial strains have excellent carbendazim degradation properties, especially *Rhodococcus* sp. Thus far, the enzyme and gene resources related to CBZ degradation are very limited. It is expected that more new genes and enzymes for carbendazim degradation will be explored further, which will facilitate further understanding of the mechanism of carbendazim microbial degradation. In addition, advanced molecular biological methods, enzyme engineering techniques and emerging materials will provide better tools for the remediation of carbendazim contamination, which lays the foundation for low-cost, efficient and complete degradation of carbendazim in the future.
